# Machine Learning Approach to Enhance the Performance of MNP-Labeled Lateral Flow Immunoassay

**DOI:** 10.1007/s40820-019-0239-3

**Published:** 2019-01-17

**Authors:** Wenqiang Yan, Kan Wang, Hao Xu, Xuyang Huo, Qinghui Jin, Daxiang Cui

**Affiliations:** 1 Department of Instrument Science and Engineering, School of Electronic Information and Electrical Engineering, Shanghai Engineering Research Center for Intelligent Diagnosis and Treatment Instrument, Key Laboratory of Thin Film and Microfabrication (Ministry of Education), Shanghai Jiao Tong University, Shanghai, 200240 People’s Republic of China; 2 School of Naval Architecture, Ocean and Civil Engineering, Shanghai Jiao Tong University, Shanghai, 200240 People’s Republic of China; 3 Department of Biomedical Engineering, JiLin Medical University, JiLin, 132013 People’s Republic of China; 4 State Key Laboratory of Transducer Technology, Shanghai Institute of Microsystem and Information Technology, Chinese Academy of Sciences, Shanghai, 200050 People’s Republic of China; 5 Faculty of Electrical Engineering and Computer Science, Ningbo University, Ningbo, 315211 People’s Republic of China

**Keywords:** Point-of-care testing, Immunochromatography test strips, Magnetic nanoparticles, Machine learning, Support vector machine

## Abstract

The use of magnetic nanoparticle (MNP)-labeled immunochromatography test strips (ICTSs) is very important for point-of-care testing (POCT). However, common diagnostic methods cannot accurately analyze the weak magnetic signal from ICTSs, limiting the applications of POCT. In this study, an ultrasensitive multiplex biosensor was designed to overcome the limitations of capturing and normalization of the weak magnetic signal from MNPs on ICTSs. A machine learning model for sandwich assays was constructed and used to classify weakly positive and negative samples, which significantly enhanced the specificity and sensitivity. The potential clinical application was evaluated by detecting 50 human chorionic gonadotropin (HCG) samples and 59 myocardial infarction serum samples. The quantitative range for HCG was 1–1000 mIU mL^−1^ and the ideal detection limit was 0.014 mIU mL^−1^, which was well below the clinical threshold. Quantitative detection results of multiplex cardiac markers showed good linear correlations with standard values. The proposed multiplex assay can be readily adapted for identifying other biomolecules and also be used in other applications such as environmental monitoring, food analysis, and national security.

**Electronic supplementary material:**

The online version of this article (10.1007/s40820-019-0239-3) contains supplementary material, which is available to authorized users.

## Highlights

- An ultrasensitive multiplex biosensor was designed to quantify magnetic nanoparticles on immunochromatography test strips.
- A machine learning model was constructed and used to classify both weakly positive and negative samples, significantly enhancing specificity and sensitivity.
- A waveform reconstruction method was developed to appropriately restore the distorted waveform for weak magnetic signals.

**Electronic supplementary material:**

The online version of this article (10.1007/s40820-019-0239-3) contains supplementary material, which is available to authorized users.

## Graphical abstract

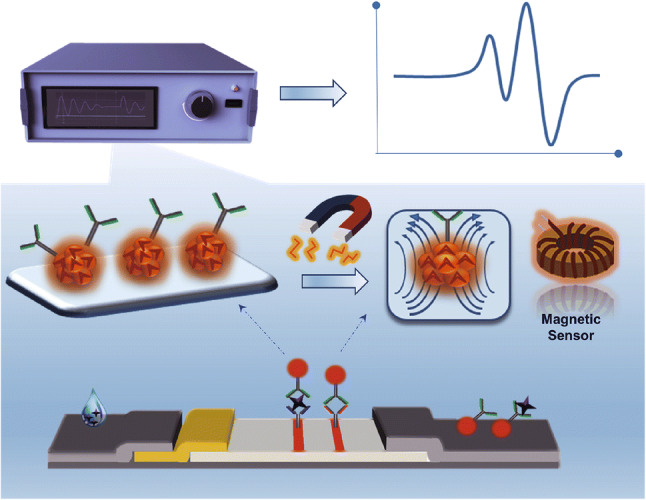

## Introduction

Acute diseases such as acute myocardial infarction (AMI) occur rapidly and can cause severe damage to health, making them a major public health problem worldwide [1, 2]. Additionally, chronic diseases must typically be detected frequently to monitor the disease state [3]. Therefore, detecting acute diseases and monitoring chronic diseases require rapid and readily accessible detection methods to detect multiplex targets simultaneously (e.g., detecting three items related to AMI). Various methods have been developed for the simultaneous analysis of diseases, such as enzyme-linked immunosorbent assay (ELISA) [4], electrochemiluminescence immunoassay (ECLIA) [5], electrochemical immunoassay [6, 7], fluorescence detection [8, 9], and label-free methods [10, 11]. However, some of these methods are time-consuming and require well-equipped facilities, complex operations, well-trained technicians, and long analysis times [12–14]. These restricted conditions limit their point-of-care testing (POCT) applications for disease detection. POCT concept provides a short assay time and simple operation and is cost-effective. Thus, POCT has been widely developed for early diagnosis over the past decade [15–20].

Immunochromatography test strips (ICTSs) are the most promising diagnostic format for POCT because they are rapid, facile, cost-effective, and user-friendly [21, 22]. ICTSs have been widely used for on-site testing, such as home pregnancy testing, environmental monitoring, and pathogen detection to ensure food safety [23–27]. However, conventional gold nanoparticle (GNP)-based ICTSs typically only provide qualitative or semiquantitative detection results with low detection sensitivity compared to traditional diagnostic methods such as the ECLIA and ELISA [23, 24, 28, 29]. Therefore, fluorophores have been developed to replace GNPs to improve the sensitivity of ICTSs [23, 30, 31]. However, the fluorophores used commonly in fluorescence ICTSs, such as fluorescent dyes, are vulnerable to photobleaching [32] and are unstable at room temperature [33]. The use of quantum dots can overcome these problems because of their favorable photostability, large molar extinction coefficients, and high fluorescent quantum yield [23, 34–36]; however, quantum dots also face some challenges, such as chemical and colloidal instability after conjugation with specific antibodies and a high auto-fluorescence background [28, 37–39]. Additionally, various ICTSs based on GNPs or fluorophores can provide quantitative results by imaging the ICTSs and analyzing the colorimetric intensity of the test zone [39–42]. However, the optical method can only analyze colorimetric intensity on the surface of the test zone, while subsurface signals are missed, reducing the signal utilization rate [43]. Further, most optical methods are based on RGB analysis or grayscale scanning, limiting their sensitivity and accuracy [44].

In the present study, a magnetic quantification method was developed by analyzing the magnetic signals from magnetic nanoparticles (MNPs) to obtain quantitative results. This approach can be employed to detect the whole test zone (three-dimensional) even when the test zone is opaque and has a high “signal-to-noise” ratio because only a weak magnetic background signal is generated by biological samples. Several magnetic quantification methods using ICTSs have been reported, such as giant magnetoresistance (GMR) sensor-based methods [45–48], tunneling magnetoresistance (TMR) sensor-based methods [49], and coil-based methods [43, 50, 51]. Particularly, Marquina et al. [47] used a GMR sensor-based method to quantitatively detect human chorionic gonadotropin (HCG), but this method showed low sensitivity, considerable noise, and a complex reuse process. Lei et al. [49] developed a TMR sensor-based method to improve the sensitivity when detecting HCG, but this method was limited because two (or more) test zones (i.e., test line and control line) had to be separated by a distance of at least 10 mm, making this method suitable for only one test zone ICTS. Several previous studies [51–54] employed a commercial magnetic assay reader (MagnaBioSciences, San Diego, CA, USA) to detect *Bacillus anthracis* spores [51], *Listeria monocytogenes* [52], the HIV-1 p24 antigen [53], and fish major allergen parvalbumin [54]. This system exhibited good sensitivity, but the position of the test zone had to be manually reset several times and the system was not user-friendly because only magnetic intensity could be detected, while other information may not be included (e.g., patient information and clinical results).

Based on these previous findings, we developed a novel approach for detecting magnetic ICTSs, which can automatically locate the positions of test zones and rapidly detect both a single-target assay strip and multiplex assay strip. We also developed a novel data processing method based on a support vector machine (SVM) classifier and custom waveform reconstruction method for weak signals, thereby greatly improving the sensitivity and accuracy. HCG was tested as a proof of concept for a single-target assay as well as three myocardial infarction markers comprising cardiac troponin I (cTnI), creatine kinase isoenzyme MB (CKMB), and myohemoglobin (Myo) for a multiplex assay, and satisfactory detection results were obtained. Quantitative analysis showed that the proposed approach is useful for rapid and quantitative detection with high accuracy and repeatability.

## Materials and Methods

### Materials

Polyvinyl chloride (PVC) plates, sample pads, conjugate pads, nitrocellulose (NC) membranes, and absorbent pads were purchased from JieNing Biological Technology Co., Ltd. (Shanghai, China). The MNPs (197 nm) were purchased from Biomag Biotechnology Co., Ltd (Jiangsu, China), and the characterization of MNPs is described in the supplementary material. The antibody (Ab) and antigen (Ag) for HCG and goat anti-mouse immunoglobulin (IgG) antibodies were purchased from Shanghai JieYi Biotechnology Co., Ltd. (Shanghai, China). The Ab and Ag for cTnI were purchased from HyTest, Ltd. (Turku, Finland) and Abcam (Cambridge, UK), respectively. The Ab and Ag for both CKMB and Myo were purchased from Fitzgerald Industries International (Acton, MA, USA).

### ICTS Format and Assay Principle

**Fig. 1:**
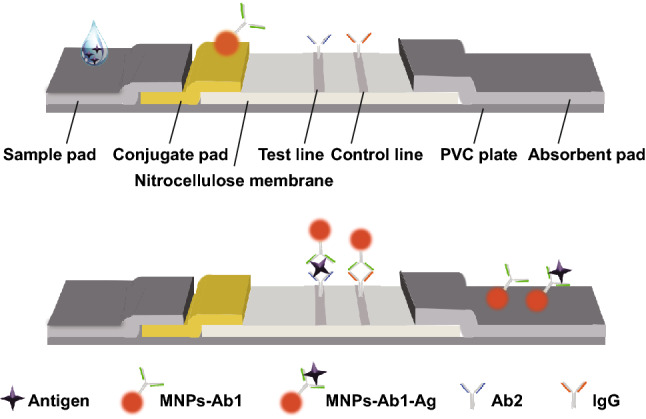
Format and assay principle of ICTS

The ICTSs were assembled with PVC plates, sample pads, conjugate pads, NC membranes, and absorbent pads, as shown in Fig. 1. The pads and NC membranes were pasted onto the PVC plates with an overlap of approximately 2 mm for each assembly unit. The assembly was cut into 3-mm strips for single-target assays and 4-mm strips for multiplex assays.

After the sample was dropped onto the sample pad, it flowed toward the absorbent pad because of capillary force. In the sandwich complex method, the Ag conjugated with MNPs-Ab (MNPs-Ab1, deposited onto the conjugate pad, preparation method is described in supplementary material) to form an immune complex (MNPs-Ab1-Ag). The complex migrated along the NC membrane until it was captured by another monoclonal Ab (Ab2) coated on the test line (TL), thereby forming a sandwich (MNPs-Ab1-Ag-Ab2). The surplus complex (MNPs-Ab1-Ag) continued to migrate along the NC membrane until it was captured by the IgG coated on the control line (CL). Therefore, magnetic signals were detected from the TL and CL. In the HCG assay, the Ab for HCG (1 mg mL^−1^) was immobilized as TL and goat anti-mouse IgG (1 mg mL^−1^) was immobilized as CL. In the multiplex cardiac markers assay, Abs for cTnI (2 mg mL^−1^), CKMB (2 mg mL^−1^), and Myo (1 mg mL^−1^) were immobilized as three TLs and goat anti-mouse IgG (1 mg mL^−1^) was immobilized as the CL.

### Development of ICTS Cartridge and Magnetic Immunoassay Reader

The ICTS cartridge was designed to accommodate the strip and protect the strip from pollution when handled, as well as to ensure that the position of the strip in the reader remained constant by pressing it tightly. The ICTS cartridge comprised two parts and was manufactured using a three-dimensional printer. The lower part mainly comprised a groove to accommodate the ICTSs and many holes for fastening. The opening in the middle of the ICTS cartridge provided a space as the testing zone. The upper part contained a square region for attaching a quick response (QR) code and inlet for sample injection.

**Fig. 2:**
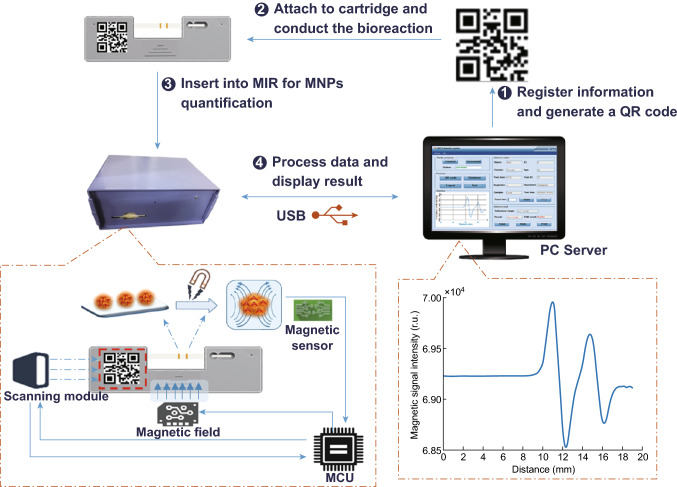
Assay procedure comprising brief working principle of MIR

In the magnetic immunoassay reader (MIR) system (Fig. 2), a QR code scanner was employed to read the QR code. A group of coils (described in the next section) was used to magnetize the MNPs and convert the magnetic intensity to voltage. Additionally, a series comprising amplifiers, digital-to-analog converter (DAC), analog-to-digital converter (ADC), field-programmable gate array (FPGA), microprogrammed control unit (MCU), and other units were used to process the voltage signal and generate digital sequential data. A step motor was used to ensure that the strip moved at a constant velocity and direction. The reader was connected to a computer via a universal serial bus (USB). First, the operating program provided an instruction to the MCU to carry out the following determination. Once the MCU received the command, the QR code scanner began to scan the QR code and the MCU activated the biosensor via the written FPGA and DAC simultaneously. As the test area moved to the ferrimagnet, the MNPs were magnetized and the magnetic signal was detected by the sensor and transformed into a voltage signal. Additionally, amplifiers were used to strengthen the acquired voltage signal. Next, an ADC was used to translate the voltage signal into a digital signal and the obtained digital signal was transmitted to the MCU via the FPGA. Ultimately, the recorded raw data and information in the QR code were transmitted to the PC via the USB.

### Development of MIR Biosensor

The MNPs had no magnetic characteristics outside an external magnetic field; otherwise, they would have attracted each other. Therefore, the MNPs only had magnetic properties when they were in a strong external magnetic field, and they rapidly lost their magnetic properties when outside the external magnetic field. A C-shaped ferromagnetic core was employed to provide an external magnetic field, and the direction of the magnetic field was perpendicular to the test strip. The strip moved at a constant velocity and direction through the magnetic field, carried by a step motor. Two coils parallel to the strip were applied to the signal induced by the magnetized MNPs, where the two coils were identical (one was used as a background reference). The coil size was similar to that of the TL, and the distance between the two coils was approximately 1 mm.

### Construction of the Program

To provide a complete disease detection system for doctors and patients, we constructed a system comprised of the following three parts: (1) Information registration and QR code generation, where the information comprised patient information (e.g., name, ID, and gender) and detected information (e.g., test item and test time). This information was stored in a database, and the patient ID was encoded and written into the QR code. The QR code was then attached to the designated area on the strip cartridge. (2) Detection conditions setting, such as the TL number (1, 2, or 3) and number of repetitions (typically from 1 to 10), followed by magnetic detection. The detection results were analyzed and presented as a detection report, which can be printed for the patient. (3) A database management system with several functions, such as multiple query information detection from the database, and modifying or exporting the information. We constructed the disease detection system based on the.NET framework (Microsoft Corporation, Seattle, WA, USA), and data were processed using a hybrid programming between the C# programming language and MATLAB (MathWorks, Inc., Natick, USA).

### Development of the Validation Card

**Fig. 3:**
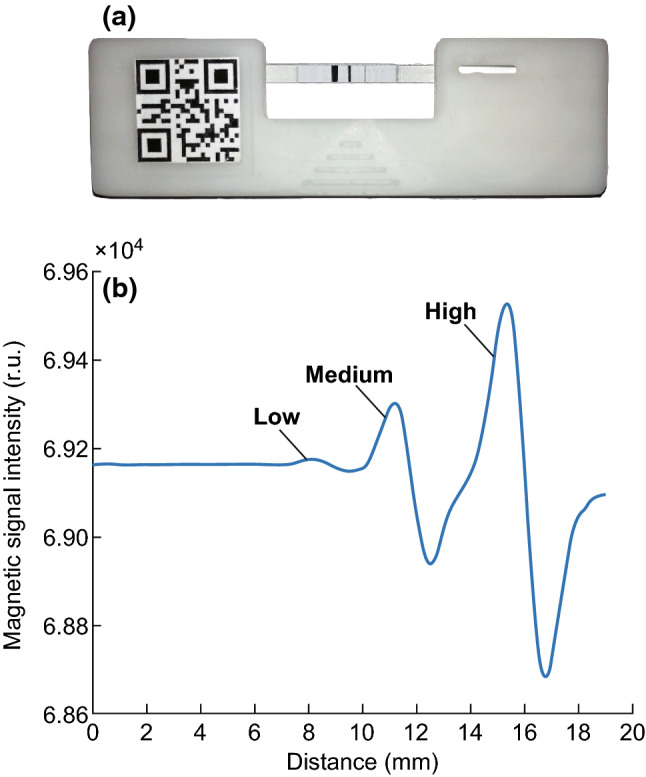
**a** Validation card with ICTS cartridge and QR code. **b** Waveform of validation card, comprising three levels of magnetic intensity: low, medium, and high

The validation card was used to confirm whether the MIR apparatus operated correctly. Therefore, we developed a validation card with three magnetic intensity levels corresponding to the magnetic intensity detected in samples diluted to low, medium, and high concentrations. Additionally, the validation card format was a strip with three lines in a similar manner to the ICTSs, such that the material used to form the lines had magnetic properties in a strong external magnetic field but rapidly lost the magnetic properties when outside of the external magnetic field. The most important requirement for the validation card was that the magnetic intensity had to remain constant under different environmental conditions (e.g., timing, temperature, and illumination). We cut slim bands from a floppy disk at different widths for use as the detection strip in the validation card and obtained three magnetic intensity levels. The validation card and its waveform are shown in Fig. 3a, b, respectively.

### Assay Procedure

As shown in Fig. 2, first, we registered the information and generated a QR code using the custom program before printing the QR code and attaching it to the designated area on the strip cartridge. Second, the sample solution was dropped onto the inlet of the strip cartridge containing a strip; the biological reaction required approximately 15 min. Third, we connected the MIR by using the custom program to control access to the MIR, and the strip cartridge with a strip was then inserted into the slot of the MIR. Fourth, we set the detection conditions and clicked the start button to start the MIR detection process, which required less than 3 min (depending on the number of repetitions) for MIR detection. The messages were stored in the QR code, and raw detection data were returned by the MIR reader. Finally, the messages and raw data were analyzed by the custom program, and the detection results were presented and stored automatically in the database. The detection report can be printed if necessary.

### Clinical Samples

This study was approved by the Medical Ethics Committee of Shanghai Jiao Tong University. All clinical samples were collected from Shanghai Ninth People’s Hospital, the affiliated hospital of Shanghai Jiao Tong University. Fifty-nine serum samples were collected from patients and stored at − 80 °C until use. The detection of serum samples is described in the supplementary material. Accurate values for the samples were obtained by ECLIA.

### Statistics

Three replicates were performed for each experiment. The average value was calculated as the result, and standard deviation of the recorded values was estimated as the error. Statistical differences were evaluated using the *t* test and considered significant when *p *< 0.05. All data were analyzed in the SPSS version 20 software (SPSS, Inc., Chicago, IL, USA), and diagrams were plotted in MATLAB 2018a and Origin Lab 8.5 (OriginLab Corporation, Northampton, MA, USA).

## Results and Discussion

### Effect of Soft Coils in the Biosensor

**Fig. 4:**
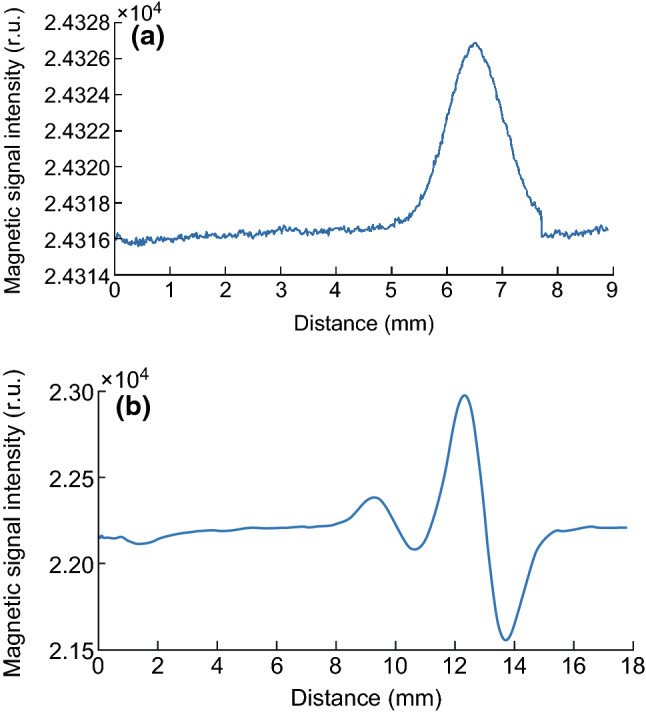
**a** Waveform for a single coil employed as the induction coil in the first version of the MIR. **b** Waveform for a pair of coils employed as the induction coil

Soft coils comprised the core of the biosensor, which was used for data acquisition. A single coil was employed as the induction coil in the first version of the MIR, but the output waveform was unsatisfactory (Fig. 4a). Only a relatively strong signal was detected in the CL, showing a peak with considerable noise. Given the weak magnetic signals in the TL, the influence of the magnetic field was also subtle, and one coil was not sufficient for detecting the signal. Thus, a pair of coils was designed to induce a change in the magnetic field due to the influence of the MNPs in the TL and CL. Parallel coils were fixed in the biosensor, with one used as the background signal. The output waveform was obtained by subtracting the signals from the two coils. The size of the coils was similar to that of the TL and CL, enabling the signals from the TL and CL to be distinguished from the background signal, with a peak and trough for each test zone in the NC membrane (Fig. 4b), which also doubled the peak-to-peak value (PPV) for the signal. Additionally, the proximate arrangement of the two coils canceled out most of the influence of other magnetic fields, such as power–frequency interference and Earth’s magnetic field.

### Data Analysis and Signal Process

**Fig. 5:**
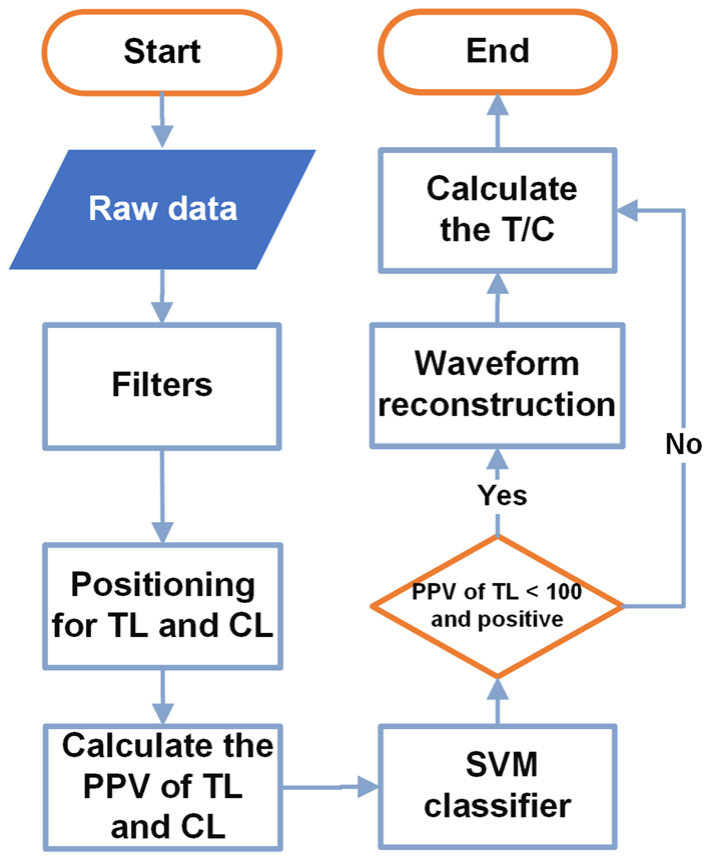
Flowchart for data processing procedure

**Fig. 6:**
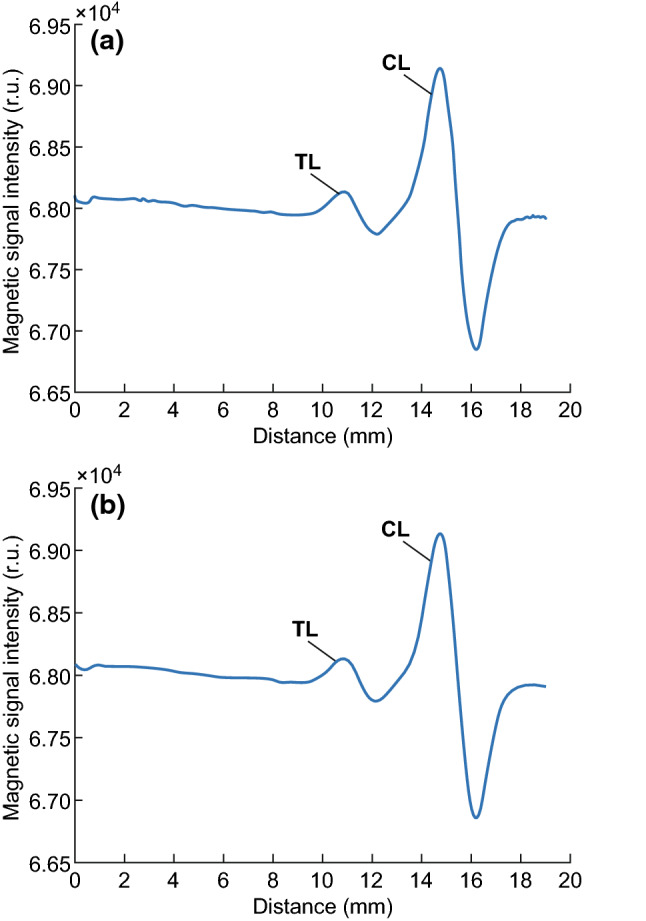
**a** Waveform of raw detection data. **b** Filtered waveform

A flowchart illustrating the data processing procedure is shown in Fig. 5. The raw data provided by the MIR apparatus comprised the digital signal, as shown in Fig. 6a, where spiked noise interfered with the magnetic signal. Therefore, filters were employed to eliminate noise. A median filter was used to eliminate the spiked noise, where it replaced a designated digit with the median of the sequence encompassing the digit, and the length of the sequence was adjusted to obtain the desired result. A moving average filter was then used to smooth the curve. The median filter eliminated the peaks and troughs of the test lines; thus, a wavelet denoising method was used to restore the desired peak values. The filtered curve is shown in Fig. 6b. Notably, a peak and a trough were present for both the TL and CL because we employed two coils for magnetic detection, as described in Sect. 2.4.

The PPV of the magnetic signal intensity for the TL and CL was used as an important index for quantitative detection. The magnetic signal intensity of the TL was weak at a low analyte concentration, whereas the magnetic signal intensity of the CL was typically high, and thus it was better to find the position of the CL first. In contrast to the magnetic assay reader for which the positions must be manually reset several times, the MIR developed in this study detected the position automatically. This is because the position of the CL was varied in different detection processes, but was always within a certain range (less than 3 mm), enabling the peak position of the CL to be found by calculating the maximum value of the data within this range. In addition, the distance between the peak and trough position was nearly constant, and so the trough position was found by calculating the minimum value of the data within a small range determined by the peak position. The PPV for the CL was obtained by subtracting the peak value and trough value.

#### Accurate Qualitative Classification

The position of the TL was selected from a small range because the distance between the TL and CL was constant on the strips. When the magnetic signal intensity of TL was not weak (as shown in Fig. 6b), the peak position and trough position were found easily for the TL according to the peak position and trough position of the CL. However, when the magnetic signal intensity of TL was weak, the waveform of TL was distorted. The noises in this system may originate from the probes, test strips, and MIR apparatus, and the frequencies of the noises were random and nonperiodic. The magnetic signal of the detection area was very weak because of the randomly distributed spots and narrow test band. Second, the target analytes were always in a complex format containing multiple components, such as broadside edges of the NC membrane during pure serum detection. Therefore, the noises cannot be eliminated by conventional denoising methods.

Powerful and robust magnetic data processing algorithms are needed to distinguish weakly positive and negative samples. There are several commonly used classification methods, including decision tree, linear discriminant analysis, *K*-nearest neighbor, Naïve Bayes, SVM, and random forest [55]. Among these, SVM is a binary classifier used for classification because it is rapid and robust when handling noisy data and is not affected by the presence of outliers [56–59]. Moreover, SVM is more suitable for classification with a small sample number compared to other classification methods. Therefore, SVM was very suitable for qualitative classification in this study.

We trained different SVM models for different analytes. Before detecting a strip, the type of analyte was registered and stored in the QR code. When detecting the strip, the QR code was decoded and the analyte type was determined. Next, a specific type of model for this type of analyte was applied for classification. Herein, low-concentration HCG strips were detected as a sample to compare the sensitivity when using the SVM classifier, as well as for visual reading and semiquantitative detection based on the ratio of the PPV for the TL and CL. Five strips were prepared for each concentration, and detection was conducted for 120 samples.

For the SVM method, the waveform for TL was used as the feature vector. Before training the SVM classifier, the importance degree of the features was sorted, enabling selection of the most valuable features for classification. After comparing the accuracies of several kernel functions, the linear kernel was selected as the kernel function for SVM in this study. To evaluate the prediction performance of the classifier, 120 detection samples were divided into five groups for fivefold cross-validation, with four groups used for training and the remaining group used for testing circularly. The concentrations of 0 and 0.25 mIU mL^−1^ were set as negative (60 samples), while the others were set as positive (60 samples). To obtain visual readings, five volunteers were invited to read the strips for the 120 results. To determine the semiquantitative detection results, we calculated the average value for each concentration and used 0.25 mIU mL^−1^ as the threshold value. The results are shown in Table 1 and indicate that the accuracy of the SVM classifier was 100% and the accuracy of the visual readings was 100% up to 2.5 mIU mL^−1^. For the semiquantitative detection method, the accuracy was 60% at 0.5 mIU mL^−1^, indicating that it could not distinguish the strips at 0.5 mIU mL^−1^. Therefore, using the SVM classifier improved the sensitivity of qualitative detection.

**Table 1:** Qualitative results using visual reading, semiquantitative detection, and SVM classifier

Group	Concentration (mIU mL^−1^)	Data number	Accuracy		
			Visual reading (%)	TL/CL (%)	SVM (%)
1	0	30	67	100	100
2	0.25	30	7	43	100
3	0.5	20	50	60	100
4	1	10	70	100	100
5	2.5	10	100	100	100
6	5	10	100	100	100
7	10	10	100	100	100

#### Restoration of the Distorted Waveform for Weak Signals

The sensitivity of qualitative detection was improved by using the SVM classifier, but the distorted waveform for the weak magnetic signal intensity in TL still influenced the accuracy of quantitative detection and a standard sinusoid peak was not produced. Therefore, we developed a waveform reconstruction method for restoring the distorted waveform when the magnetic signal intensity was weak. Briefly, the original peak and trough position were identified for the TL and CL, and then their PPV was calculated (labeled as pT and pC, respectively). Typically, pT was less than 100, while pC ranged from 2000 to 3000. The waveform for the CL was typically normative and thus could be used as a reference template. We then scaled down the value as a reconstructed waveform for the TL within a range for pT from − 10 to + 10, where we assumed that the range was *r*. Next, the waveform for the TL was compared to the scaled waveform for the CL as follows.

1. The waveform for the TL was determined by setting the trough of the TL as the last point of the waveform, before selecting *m*-1 sequential points ahead of the trough for the TL. *m* was a constant integer and identified in advance based on the waveforms of the strong signal. A similar method was used to obtain *m* sequential points from the scaled waveform for the CL.
2. The difference between the waveform for the TL and scaled waveform for the CL was calculated using Eq. 1, where *T*_i_ is a random point among *m* points of the waveform for the TL and *C*_i_ is the corresponding point among m points of the waveform for the scaled CL. The algorithm was applied to each scaled waveform in the range *r*.
  1 $${\text{Difference}} = \sum \limits_{m} \left({T_{\rm i} - C_{\rm i}} \right)^{2}$$
3. Finally, the minimum difference in range r was calculated, and the corresponding waveform was the proximate wave rather than the original TL.

**Fig. 7:**
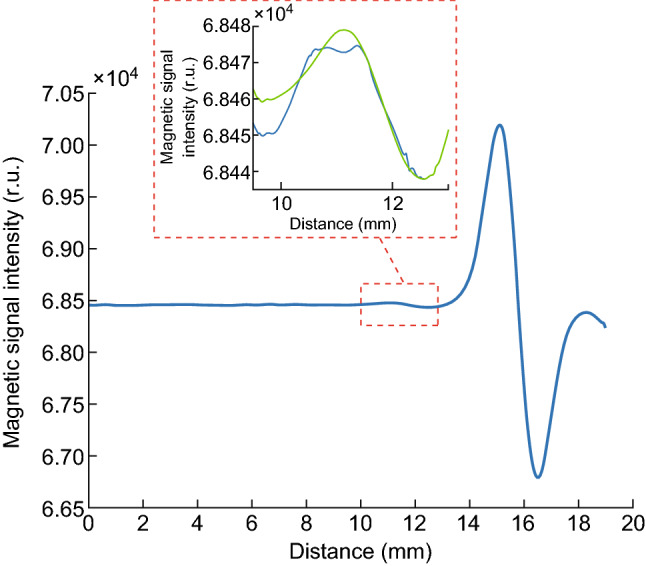
Results of waveform reconstruction (in the small diagram). Blue line is the distorted waveform, and green line is the reconstructed waveform. (Color figure online)

The reconstructed waveform is shown in Fig. 7; in the small diagram, the blue line is the distorted waveform and the green line is the reconstructed waveform. The results showed that the reconstructed waveform had restored the distorted waveform. The waveform for the TL was normalized based on the reconstructed waveform for the TL and the original waveform for the CL, with noise beyond the test area eliminated.

#### Performance and Sensitivity of Magnetic ICTS

**Fig. 8:**
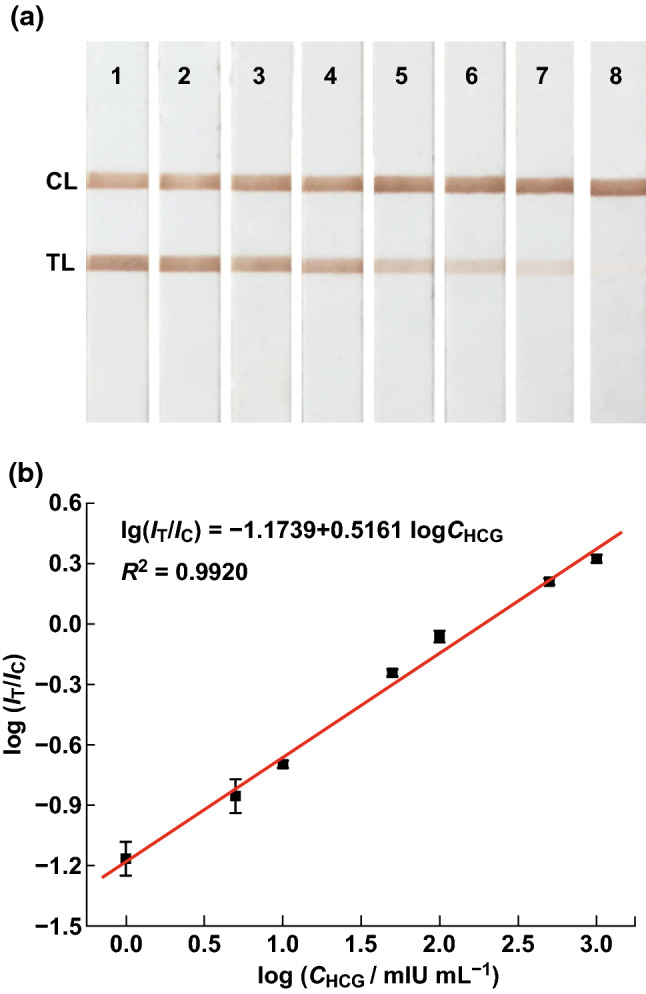
**a** HCG strips used for standard curve. Concentrations from 1 to 8 were 1000, 500, 100, 50, 10, 5, 1, and 0 mIU mL^−1^. **b** Standard curve for HCG strips

It is well known that a higher concentration of analyte leads to a stronger magnetic signal intensity, except in a saturation situation; thus, examining their relationship can facilitate quantitative detection. We selected the ratio of PPVs for the TL and CL rather than their areas because the ratio of PPVs was equal to the ratio of the areas after the waveform reconstruction process and was much easier to calculate directly. In addition, using pT/pC rather than only pT eliminated the influence of the difference between the strips [23, 28, 36, 37, 42]. The standard curve was generated by detecting HCG antigen at 1, 5, 10, 50, 100, 500, and 1000 mIU mL^−1^ (with three strips for each concentration); the strips are shown in Fig. 8a. Each strip was evaluated three times under the same conditions. The average value and standard deviation based on the tests were calculated as the result and error, respectively. The limit of detection (LOD) was calculated based on the criterion of three times the standard deviation for the Ag-free negative control [60]. We observed a linear relationship between the T/C ratio and concentration HCG using the logarithmic form (Fig. 8b), where the equation is: log (*I*_T_/*I*_C_) = –1.1739 + 0.5161 × log *C*_HCG_ (*R*^2^ = 0.9920). The dynamic range was 1–1000 mIU mL^−1^ and the LOD was 0.014 mIU mL^−1^. The results of comparison of different detection methods for the antigen HCG are shown in Table 2, which showed a wide linear range with a lower LOD, and a short time was required for detection compared to some other reported methods.

**Table 2:** Comparison of different detection methods for HCG

Measurement method	Linear range (mIU mL^−1^)	Detection limit	Detection time (min)	References
Fluorescent method	50–20,000	20 mIU mL^−1^	30	[61]
Chemiluminescence immunoassay	0.1–10	0.06 mIU mL^−1^	180	[62]
Surface-enhanced Raman scattering	0.5–200	0.18 mIU mL^−1^	35	[63]
GNPs ICTS	10–120	2.3 mIU mL^−1^	15	[41]
GNPs ICTS (with gold enhancement)	–	0.3 mIU mL^−1^	40	[64]
CdSe/ZnS QDs ICTS	–	0.5 mIU mL^−1^	10	[65]
UCPs ICTS	–	100 pg mL^−1^	15	[66]
MNPs ICTS	–	0.31 ng mL^−1^	15	[67]
MNPs ICTS	1–1000	0.014 mIU mL^−1^	20	This work

### Accuracy Test

The accuracy can indicate systematic errors in a system and was calculated by comparing the concentrations measured with different spiking concentrations. The system accuracy was tested by detecting five concentrations (1, 10, 50, 200, and 500 mIU mL^−1^) of HCG standard solutions (with three strips for each concentration), where each strip was inserted into the reader 10 times and the average value and standard deviation were calculated for each concentration. System accuracy was evaluated by calculating recovery as the ratio of the average detected concentration and standard concentration. The detection results are shown in Table 3, with the detected concentrations shown as the average values and double standard deviations. The recovery using this system ranged from 92.80 to 115.63%, indicating good accuracy.

**Table 3:** Accuracy of the system at different concentrations

Group	Standard concentration (mIU mL^−1^)	Detected result (mIU mL^−1^)	Recovery (%)
1	1	1.10 ± 0.16	110.08
2	10	9.28 ± 0.40	92.80
3	50	57.81 ± 3.79	115.63
4	200	211.46 ± 7.32	106.18
5	500	477.23 ± 16.50	95.45

### Repeatability Analysis

The repeatability represents precision under the same assay conditions, i.e., same analyst, detection instrument, and reagents. Repeatability can indicate random errors and variations caused by different settings (e.g., location of TL), which may influence the detection results. Before conducting an assay, the validation card should be detected first to confirm that the system functions correctly. In this study, system repeatability was tested by detecting three concentrations (5, 50, and 500 mIU mL^−1^) of HCG standard solutions (with a single strip for each concentration), where each strip was inserted into the reader 10 times. System repeatability was evaluated by calculating the coefficient of variation (CV) as the ratio of the standard deviation relative to the average detected value. A lower CV value indicates better stability and repeatability of the system. At the three HCG concentrations of 5, 50, and 500 mIU mL^−1^, the CV values were 2.92%, 1.54%, and 1.09%, respectively. The maximum CV value for the system was 2.92%, indicating the good repeatability of the system, and thus the detection results obtained using the reader and algorithm developed in this study were stable.

### Multiplex Assay of Clinical Cardiac Markers

**Fig. 9:**
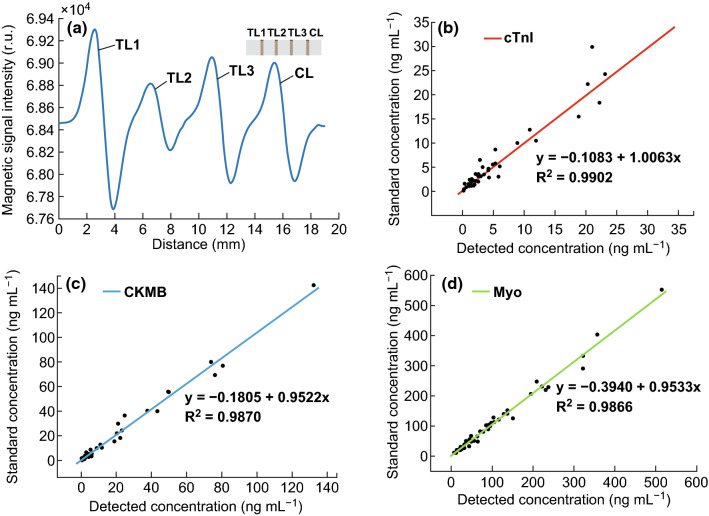
**a** Multiplex line strip and its waveform for detecting cTnI (TL1), CKMB (TL2), and Myo (TL3) simultaneously. Correlation between the results obtained by clinical ECLIA methods and this platform for **b** cTnI, **c** CKMB, and **d** Myo detection in serum samples

To evaluate the capacity for detection in a multiplex assay, a multiplex line strip was used with a CL and three TLs to detect cTnI, CKMB, and Myo simultaneously. The multiplex strip and its waveform are shown in Fig. 9a. The peaks and troughs for the CL and the three TLs represented an intact sinusoid, and the PPV for the TLs changed as the analyte concentrations were changed. The data processing method described in Sect. 3.2 can also be applied in the multiplex assay. Briefly, the CL remained in a constant range and three TLs were detected according to the distance between them and the CL. The multiplex assay strips were fabricated with constant dimensions, and line positions in the test area were confirmed to avoid mixing. Images of the test strips with different concentrations of multiplex markers are shown in supplementary material. Fifty-nine clinical samples of cardiac markers (cTnI, CKMB, and Myo) were detected to evaluate the analytical performance of the multiplex assay. The linear dependence between our method and the ECLIA method was analyzed (Fig. 9b–d), and the slopes of the regression equations and regression coefficient were all close to 1, demonstrating a good linear correlation between the two methods.

## Conclusion

In this study, we developed a user-friendly, sensitive, and rapid diagnostic method for detecting magnetic signals on ICTSs. This approach rapidly detected weak magnetic signals (10^−7^–10^−4^ Oe) on test strips, which were analyzed based on an SVM model and waveform reconstruction method automatically. Analytical performance was evaluated by detecting HCG and the simultaneous detection of three cardiac markers (cTnI, CKMB, and Myo), where HCG was quantitatively detected in the range of 1–1000 mIU mL^−1^ with a detection limit of 0.014 mIU mL^−1^. Furthermore, the detection results for the clinical samples of cardiac markers showed good linear correlations with the standard values. Therefore, this approach can detect both single and multiplex targets and has potential applications such as POCT in in vitro diagnostics, environmental monitoring, food analysis, and national security.

## Electronic supplementary material

Below is the link to the electronic supplementary material.

Supplementary material 1 (PDF 230 kb)
