# Supplementary material for: Machine Learning Approach to Enhance the Performance of MNP-Labeled Lateral Flow Immunoassay
Source: Nanomicro Lett. 2019 Jan 17;11:7. doi: 10.1007/s40820-019-0239-3 (PMC7770769; doi:10.1007/s40820-019-0239-3)
Supplement: Supplementary file 1 — Supplementary material 1 (PDF 230 kb) [file 40820_2019_239_MOESM1_ESM.pdf]

Supporting Information for

Machine Learning Approach to Enhance the Performance of MNP-Labeled Lateral Flow Immunoassay

Wenqiang Yan[1, +], Kan Wang[1, *, +], Hao Xu[2], Xuyang Huo[3, *], Qinghui Jin[4, 5], Daxiang Cui[1, *]

[1]Department of Instrument Science and Engineering, School of Electronic Information and Electrical Engineering, Shanghai Jiao Tong University, Shanghai Engineering Research Center for Intelligent diagnosis and treatment instrument, Key Laboratory of Thin Film and Microfabrication (Ministry of Education), Shanghai 200240, People's Republic of China

[2]School of Naval Architecture, Ocean & Civil Engineering, Shanghai Jiao Tong University, Shanghai 200240, People's Republic of China

[3]Department of Biomedical Engineering, JiLin Medical University, JiLin 132013, People's Republic of China

[4]State Key Laboratory of Transducer Technology, Shanghai Institute of Microsystem and Information Technology, Chinese Academy of Sciences, Shanghai 200050, People's Republic of China

[5] Faculty of Electrical Engineering and Computer Science, Ningbo University, Ningbo 315211, People's Republic of China

[+]These authors have contributed equally to this work

*Corresponding authors. E-mail: wk_xa@163.com (Kan Wang), huoxuyang@jlmu.cn (Xuyang Huo), dxcui@sjtu.edu.cn (Daxiang Cui)

S1 Preparation of Immunomagnetic Probes

Herein, the preparation procedures of immunomagnetic probes for HCG, cTnI, CKMB and Myo determination were almost same. In brief, 1 mg MNPs was dissolved by 250 μL 50 mM pH 5.5 MES buffer with 10min ultrasonication. Following, EDC (2.0 mg) and NHS (2.0 mg) were added into above-obtained solution for 30 min –COOH activation at the surface of MNPs. After remove excess chemical reagents by magnetic separation, 50 μg HCG, 80 μg cTnI, 60 μg CKMB and 50 μg Myo antibodies were respectively reacted with above-activated MNPs in 5 mM pH 9 borate solution (BS) buffer for 3 h at 37 °C under rotation. Following magnetic washing steps, 250 μL 5 mM BS (pH 7.4) containing 5% BSA was added into above-systems in respective to block the nonspecific sites of the MNPs. Ultimately, the immunomagnetic probes were resuspended in buffer and store at 4 °C for further use.

S2 Characterization of Magnetic Nanoparticles

The structure and morphology of MNPs have been characterized by transmission electron microscopy (TEM). As showed in Fig. S1, the magnetic nanoparticles presented spherical structure and uniform size with an average diameter of 197 nm. In addition, an obvious polymer layer on the surface of magnetic nanoparticles could be observed, indicating favorable biocompatibility, excellent stability and numerous carboxyl groups of used nanoparticles.

S3 Detection of Multiplex Cardiac Markers

59 serum samples were detected to evaluate the multiplex detection ability of this system. First, the collected serum samples were diluted with a working buffer of 1/1 (v/v),which was prepared by our group and composed of 10 mM PBS pH 7.4 buffer, 0.1% glycine, 2% BSA, 0.1% Tris base, and et al.. Second, 80 μL of pretreated serum was mixed with 4 μL of cTnI, CKMB, and Myo probes (total 12 μL) for 10 min. Then, the probes were enriched by an external magnet, and 80 μL of the supernatant was removed. After that, the probes were dispersed and dripped onto the conjugate pad. Next, 80 μL of the working buffer was dripped onto the sample pad to facilitate the migration of probes. After 10 min, another 30 μL of working buffer was added to wash the MNPs remain in the NC membrane (not test lines). Finally, the strip was assembled to a cartridge and detected by the MIR apparatus, and the target concentrations of serum samples were estimated according to the standard curve obtained by the antigen solutions in the working buffer.

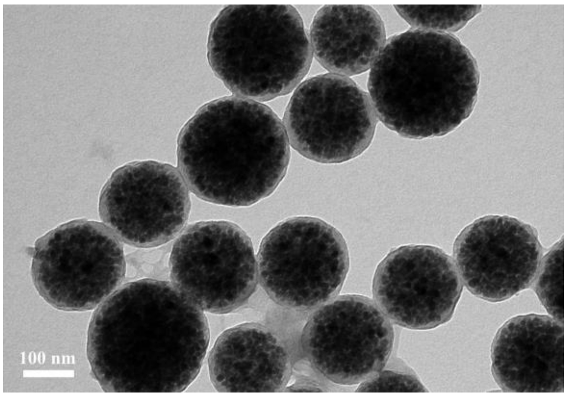

Fig. S1 TEM image of magnetic nanoparticles

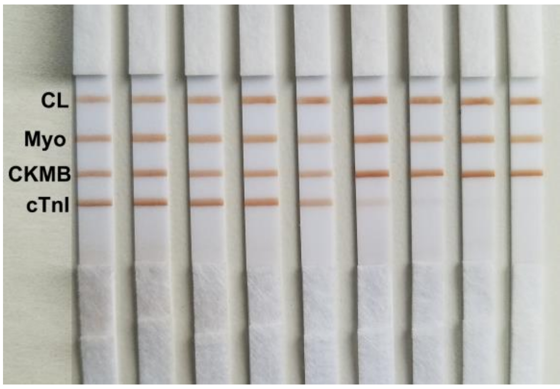

Fig. S2 Different concentration (250, 100, 50, 10, 5, 1, 0.5, 0.1, and 0 ng mL^{-1}) of cTnI samples in working buffer, with constant concentration of CKMB (100 ng mL^{-1}) and Myo (100 ng mL^{-1})

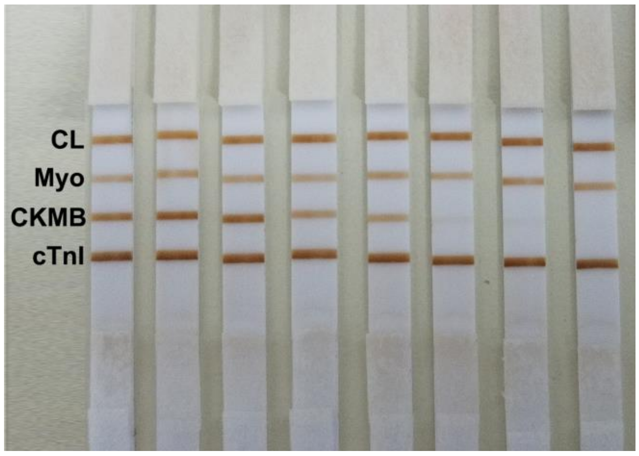

Fig. S3 Different concentration (250, 100, 50, 10, 5, 1, 0.5, and 0 ng mL^{-1}) of CKMB samples in working buffer, with constant concentration of cTnI (100 ng mL^{-1}) and Myo (100 ng mL^{-1})

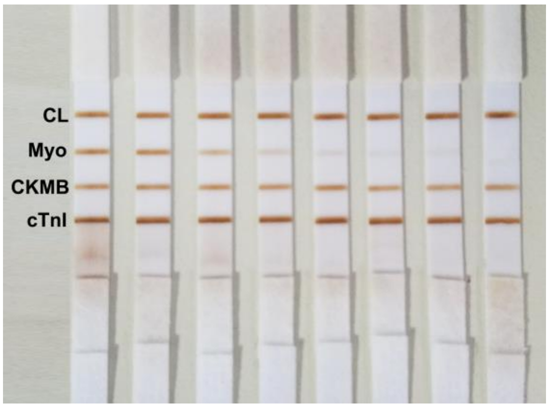

Fig. S4 Different concentration (1000, 500, 100, 50, 10, 5, 1, and 0 ng mL^{-1}) of Myo samples in working buffer, with constant concentration of cTnI (100 ng mL^{-1}) and CKMB (100 ng mL^{-1})
